# Differential Expression of E2F Transcription Factors and Their Functional and Prognostic Roles in Human Prostate Cancer

**DOI:** 10.3389/fcell.2022.831329

**Published:** 2022-04-21

**Authors:** Zhaodong Han, Rujun Mo, Shanghua Cai, Yuanfa Feng, Zhenfeng Tang, Jianheng Ye, Ren Liu, Zhiduan Cai, Xuejin Zhu, Yulin Deng, Zhihao Zou, Yongding Wu, Zhouda Cai, Yuxiang Liang, Weide Zhong

**Affiliations:** ^1^ Department of Urology, Guangdong Key Laboratory of Clinical Molecular Medicine and Diagnostics, Guangzhou First People’s Hospital, School of Medicine, South China University of Technology, Guangzhou, China; ^2^ Department of Urology, Affiliated Dongguan Hospital, Southern Medical University, Dongguan, China; ^3^ Department of Urology, Guangdong Key Laboratory of Urology, The First Affiliated Hospital of Guangzhou Medical University, Guangzhou Medical University, Guangzhou, China; ^4^ Department of Urology, The Fifth Affiliated Hospital of Guangzhou Medical University, Guangzhou, China; ^5^ Department of Urology, Affiliated Cancer Hospital & Institute of Guangzhou Medical University, Guangzhou Medical University, Guangzhou, China; ^6^ Department of Andrology, Guangzhou First People’s Hospital, School of Medicine, South China University of Technology, Guangzhou, China

**Keywords:** prostate cancer, E2F transcription factors, prognosis, biochemical recurrence, bioinformatic analysis

## Abstract

Given the tumor heterogeneity, most of the current prognostic indicators cannot accurately evaluate the prognosis of patients with prostate cancer, and thus, the best opportunity to intervene in the progression of this disease is missed. E2F transcription factors (E2Fs) have been reported to be involved in the growth of various cancers. Accumulating studies indicate that prostate cancer (PCa) carcinogenesis is attributed to aberrant E2F expression or E2F alteration. However, the expression patterns and prognostic value of the eight E2Fs in prostate cancer have yet to be explored. In this study, The Cancer Genome Atlas (TCGA), Kaplan–Meier Plotter, Metascape, the Kyoto Encyclopedia of Genes and Genomes (KEGG), CIBERSORT, and cBioPortal and bioinformatic analysis were used to investigate E2Fs in patients with PCa. Our results showed that the expression of E2F1–3, E2F5, and E2F6 was higher in prostate cancer tissues than in benign tissues. Furthermore, elevated E2F1–3 and E2F5 expression levels were associated with a higher Gleason score (GS), advanced tumor stage, and metastasis. Survival analysis suggested that high transcription levels of E2F1–3, E2F5, E2F6, and E2F8 were associated with a higher risk of biochemical recurrence. In addition, we developed a prognostic model combining E2F1, E2F6, Gleason score, and the clinical stage that may accurately predict a biochemical recurrence-free survival. Functional enrichment analysis revealed that the E2F family members and their neighboring genes were mainly enriched in cell cycle-related pathways. Somatic mutations in different subgroups were also investigated, and immune components were predicted. Further experiments are warranted to clarify the biological associations between Pca-related E2F family genes, which may influence prognosis via the cell cycle pathway.

## Introduction

Prostate cancer (PCa) represents the second most prevalent solid tumor in men worldwide (Gandaglia et al., 2021). In the United States, 2021 cancer statistics revealed that PCa was the cancer with the highest incidence in men (248,530 cases, or 26% of all cases) and ranked second in mortality rate (34,130 cases, or 11% of all cases) (Siegel et al., 2021). Although androgen deprivation therapy does benefit patients with PCa, nearly all men will inevitably develop castration resistance and have a poor prognosis (Xu and Qiu, 2019). Thus, prognostic tools are vital for screening these patients, which can allow us to intervene early and prolong the progression to castration-resistant prostate cancer (CRPC).

Prostate-specific antigen (PSA), Gleason score, and the clinical stage are commonly used to determine the prognosis of individuals with PCa. However, most single diagnostic or prognostic markers have limitations. For example, some studies have shown that PSA has weak specificity for clinically significant [grade group (GG) > 2] prostate cancer (Venderbos and Roobol, 2011; Eyrich et al., 2021). Therefore, the identification of combination and reliable predictive biomarkers is urgently required for an early diagnosis and precise prognosis and may help develop novel molecule-targeted therapeutic strategies for PCa.

E2F transcription factors (E2Fs) are a group of transcription factors (TFs) that are extensively expressed in numerous tissues and organs in higher eukaryotes (Attwooll et al., 2004). Eight E2Fs have been discovered in mammalian cells thus far, and they were named in order of discovery, that is, E2F1-8 (Iaquinta and Lees, 2007). E2Fs control many molecular activities, such as cellular proliferation, differentiation, DNA repair, cell cycle control, and cell death, and have been studied across cancers (Chen et al., 2009). E2Fs have been shown to be potential indicators for predicting prognosis in breast cancer, lung cancer, and ovarian cancer (Sun et al., 2018; Sun et al., 2019; Zhou et al., 2019). In our previous study, we found that E2F1 promoted the invasion and migration of prostate cancer cells by regulating CD147 and, importantly, that overexpression of E2F1 predicted a poor prognosis of human PCa (Liang et al., 2016). Additionally, E2F1 may be involved in the transformation of lethal prostate cancer (Rodriguez-Bravo et al., 2018; Liu et al., 2019). As a transcription factor, E2F3 directly targets IL-6 signaling and is involved in prostate tumorigenesis (Libertini et al., 2012), and dysregulation of the E2F5/p38/SMAD3 circuitry has been found to reinforce the protumorigenic switch of TGFβ signaling in prostate cancer (Majumder et al., 2016). Additionally, E2F7, regulated by miR-30c, inhibits apoptosis and promotes the cell cycle of prostate cancer cells (Wang et al., 2020). However, little is known about the effects of E2F2, E2F4, E2F6, and E2F8 on prostate pathogenesis and cancer progression. Moreover, the majority of E2Fs have yet to be fully characterized in PCa in terms of expression levels, genetic changes, biological roles, molecular processes, and prognostic significance. Thus, identification of the underlying mechanisms of E2F-mediated tumor-related genes as predictive biomarkers might provide novel therapeutic options for PCa.

In this study, we used different public datasets of high-throughput transcriptome sequencing data, and a comprehensive analysis of the relationships between the eight E2F transcription factors and the development and progression of PCa was conducted.

## Methods

### Ethical Statement

This study was approved by the Ethics Committee of Guangzhou First People’s Hospital, School of Medicine, South China University of Technology, Guangzhou, P.R. China.

### TCGA and cBioPortal Analysis

A dataset of 498 prostate cancer patients encompassing 499 prostate cancer tissues and matched clinical information was downloaded from the TCGA public database (TCGA-PRAD, The Cancer Genome Atlas-Prostate Adenocarcinoma: https://www.cancer.gov/tcga), and E2F mRNA expression and clinical significance in prostate cancer were investigated.

The Memorial Sloan Kettering Cancer Center’s cBioPortal (http://www.cbioportal.org/) for cancer genomics provides information for the integrative analysis of complicated cancer genomes and clinical profiles from the TCGA database (Hu et al., 2014).

The frequency of E2F family gene alterations (amplifications, deep deletions, and missense mutations), copy number variants determined with the pGenomic Identification of Significant Targets in Cancer (GISTC), and mRNA expression z scores (RNA Seq V2 RSEM) were all assessed using the cBioPortal for TCGA Prostate Adenocarcinoma (TCGA, Firehose Legacy, 499 samples).

### Development and Validation of the E2F Prognostic Model

Univariate Cox regression analysis was performed based on E2F family genes and clinical variables (Gleason score and T stage) through the R package “survival” to evaluate the correlation between these factors and biochemical recurrence (BCR) status in the TCGA-PRAD cohort, and statistically significant factors (*p* < 0.05) were selected for the stepwise Cox regression analysis. A forest plot was generated to visualize the results of the multivariate analysis.

Based on the significant prognostic factors from the multivariate Cox regression analysis, a nomogram for BCR was developed with the TCGA-PRAD dataset with the R package “rms” to predict the 3-year and 5-year relapse-free survival probability. The concordance index (C-index) evaluates the consistency between a nomogram’s prediction results and the actual observed results. A calibration curve was used to show the difference between the predictions of the model and the real outcomes.

A risk score was calculated by multiplying the mRNA expression level of each BCR-related E2F gene with the regression coefficient (β): $Risk\;score=\;\sum_{i=1}^{n}\beta_{i}*\left(\exp ression\;of\;mRNA_{i}\right)$ . According to the median risk score, the patients were divided into two groups: the high-risk group and the low-risk group. The survival differences between the two groups were compared by the log-rank test. A receiver operating characteristic (ROC) analysis was performed to evaluate the predictive ability of the risk score.

### Somatic Mutation Analysis

Based on the median values of gene expression, PCa patients were divided into a high E2F expression group and a low E2F expression group. Mutation data of each group were analyzed and visualized using the “maftools” package. Mutation information for each gene in each sample was demonstrated by waterfall plots.

### Immune Cell Infiltration Analysis

CIBERSORT (Cell-type Identification by Estimating Relative Subsets Of RNA Transcripts; http://cibersort.stanford.edu), a deconvolution analytical tool, can be used to calculate the abundance of immune cells in a gene expression matrix by linear support vector regression (Newman et al., 2015). To evaluate the effect of the E2F expression level on immune cells, the abundance of the 22 immune cell subtypes in the TCGA-PRAD dataset were obtained via the “CIBERSORT” R package. The infiltration levels in the high expression group and low expression group were visualized by the “ggplot” R package.

### Spearman and STRING

In the TCGA-PRAD cohort, the Spearman correlation coefficients between E2F family genes and other coding genes were calculated. The genes that had absolute correlation coefficient values in the top 20 and had *p* values less than 0.05 were considered co-expressed genes of E2Fs.

STRING (https://string-db.org/) is a database for predicting protein–protein interactions. The 103 closest interacting proteins were chosen for investigation. In our research, we selected only interactions verified by databases, experiments, and co-expression sources.

### Functional Enrichment Analysis

Metascape (http://metascape.org) is a gene annotation and analysis tool. It was utilized to conduct pathways and process enrichment analyses of E2Fs and surrounding genes that were shown to be significantly linked with E2F expression changes. The pathways and process enrichment analyses were carried out with the following ontology sources: GO Biological Processes, Reactome Gene Sets, CORUM, WikiPathways, and Canonical Pathways. All genes in the genome were used as the enrichment background. To better understand the relatedness between terms, a subset of enriched terms was chosen and displayed as a network plot, with terms having a similarity greater than 0.3 linked by edges. The molecular complex detection (MCODE) algorithm was applied to identify densely-connected network components.

### Visualization of Pathways

Database for Annotation, Visualization, and Integrated Discovery (DAVID) (https://david.ncifcrf.gov/) supplies investigators with a complete collection of functional annotation tools to comprehensively analyze the biological function of a large number of genes. DAVID was used to investigate and visualize the enriched KEGG pathways of the E2F family members and their adjacent genes.

### Statistical Analyses

All statistical analyses were performed by GraphPad Prism 8 (GraphPad Software, United States). Continuous variables are shown as the means ± standard deviations. Student’s t test or analysis of variance was used to determine the statistical significance of quantitative data (ANOVA). For the survival analysis, the Kaplan-Meier method or Cox proportional hazards regression model was utilized. Differences were considered statistically significant when the *p* value was less than 0.05.

## Results

### Description of Transcriptional Levels of E2Fs in Patients With Prostate Cancer

**FIGURE 1:**
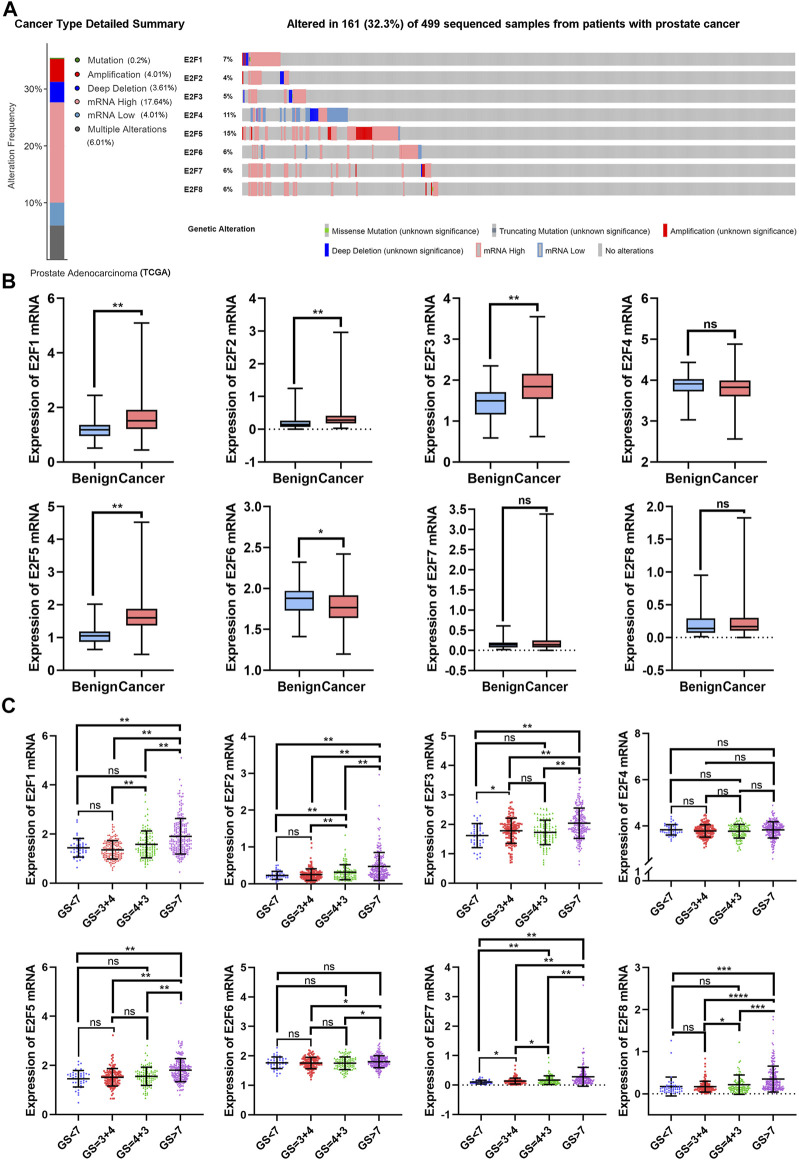
The transcription levels of E2F family members in prostate cancer patients. **(A)** Analysis of E2F gene family alterations in prostate cancer (cBioPortal). **(B)** Comparison of mRNA expression between benign tissues and prostate cancer tissues. **(C)** Analysis of the relationship between E2F expression and Gleason score in PCa patients. **p < 0.01, *p < 0.05, ns: *p* > 0.05.

With the cBioPortal web tool, the alterations of E2F family members in 499 samples from patients with prostate adenocarcinoma in The Cancer Genome Atlas (TCGA) database were detected. Aberrant E2F expression, including mutation, amplification, deep deletion, mRNA up-regulation, mRNA down-regulation, and multiple alterations, occurred in 161 samples (32.3%), as shown in Figure 1A. Up-regulation of mRNA was observed in the majority of these samples with alterations (17.64%).

Next, the TCGA-PRAD dataset from the TCGA database was used to examine the transcriptional levels of E2Fs in the prostate tissue (Figures 1B,C). E2F1 expression was higher in PCa than in benign tissues (benign vs. cancer = 1.18 ± 0.05 vs. 1.64 ± 0.03, *p* < 0.01), and a high E2F1 expression was associated with higher Gleason scores (GS < 7: 1.44 ± 0.06, GS = 4 + 3: 1.58 ± 0.05, GS > 7: 1.91 ± 0.05).

E2F2 was up-regulated in PCa in comparison to the benign tissue (benign vs. cancer = 0.19 ± 0.03 vs. 0.35 ± 0.01, *p* < 0.01). The expression of E2F2 was also shown to be up-regulated in PCa with higher Gleason scores (GS < 7: 0.23 ± 0.02, GS = 4 + 3: 0.32 ± 0.02, GS > 7: 0.47 ± 0.03).

Compared to the benign tissue, PCa tissue showed an up-regulated expression of E2F3 (benign vs cancer = 1.49 ± 0.05 vs. 1.86 ± 0.02, *p* < 0.01), and a higher expression of E2F3 was also related to higher Gleason scores (GS < 7: 1.62 ± 0.06, GS = 4 + 3: 1.73 ± 0.04, GS > 7: 2.04 ± 0.04). Likewise, similar trends were observed in E2F5 (benign vs cancer = 1.06 ± 0.04 vs. 1.64 ± 0.02; GS < 7: 1.46 ± 0.05, GS = 3 + 4: 1.52 ± 0.03, GS > 7: 1.81 ± 0.03).

Regarding E2F6, the expression was observed to be down-regulated in PCa compared to benign tissues (benign vs. cancer = 1.85 ± 0.03 vs. 1.78 ± 0.01, *p* < 0.05). No difference was observed between PCa patients with a Gleason score<7 and those with a Gleason score>7 (GS < 7: 1.77 ± 0.03 GS > 7: 1.80 ± 0.01). No significant difference in transcriptional levels between PCa and benign tissues was found for E2F4, E2F7, or E2F8.

### Relationship Between mRNA Levels of E2Fs and Clinicopathological Parameters and Prognosis in Prostate Cancer

**FIGURE 2:**
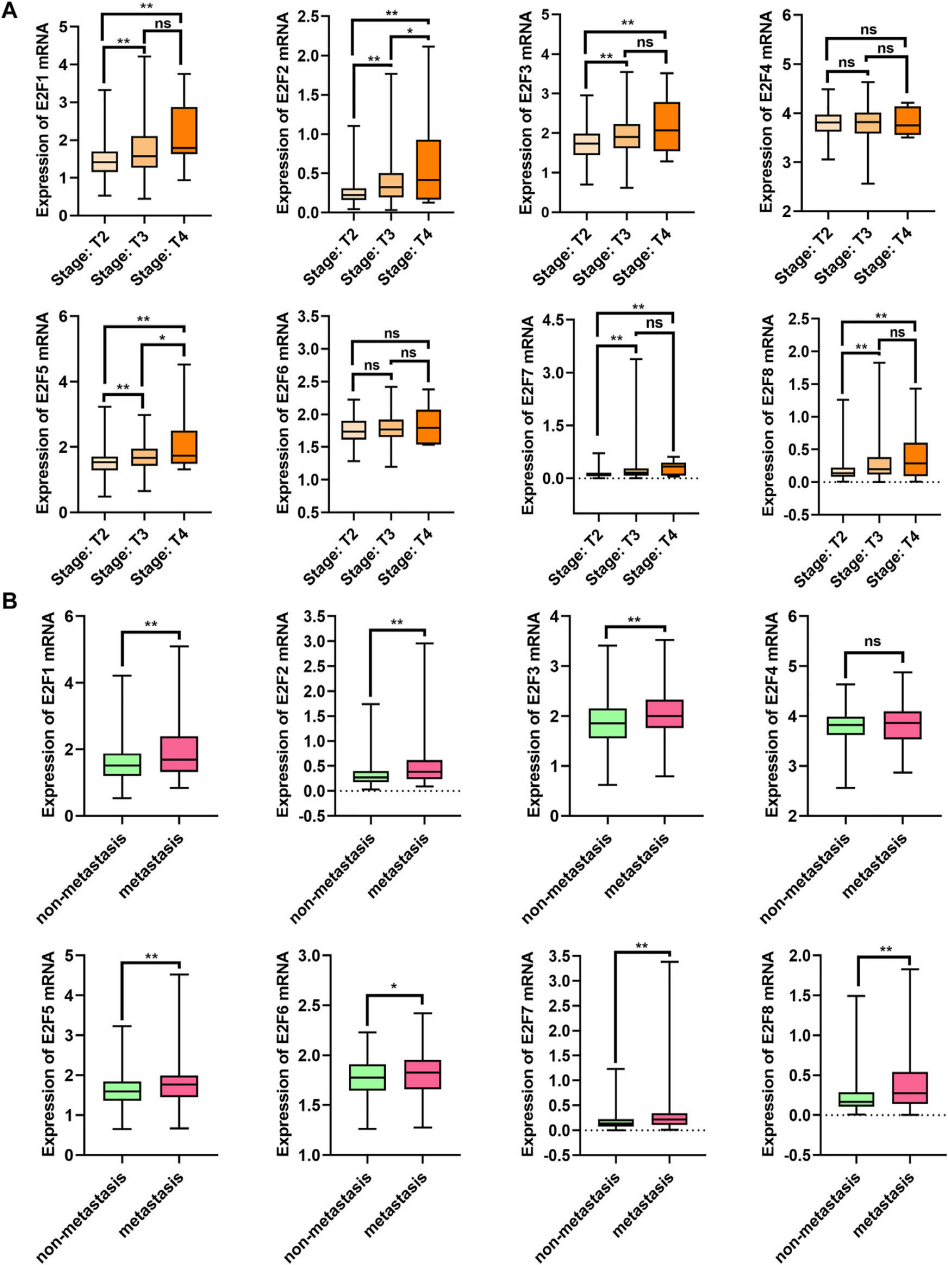
Correlation between E2F mRNA levels and clinicopathological features. **(A)** Correlation between mRNA levels and tumor stage in PCa patients. **(B)** Transcription levels in PCa patients with metastasis and without metastasis. **p < 0.01, *p < 0.05, ns: *p* > 0.05.

The relationship between E2Fs mRNA expression and clinicopathological parameters in patients with PCa was investigated with the TCGA-PRAD dataset (Figure 2). High expression levels of E2F1, E2F2, E2F3, and E2F5 mRNA were related to advanced clinical stages, while the levels of E2F4, E2F6, E2F7, and E2F8 mRNA did not change substantially (Figure 2A). All members except E2F4 showed increased expression levels in PCa tissues with metastasis (Figure 2B).

**FIGURE 3:**
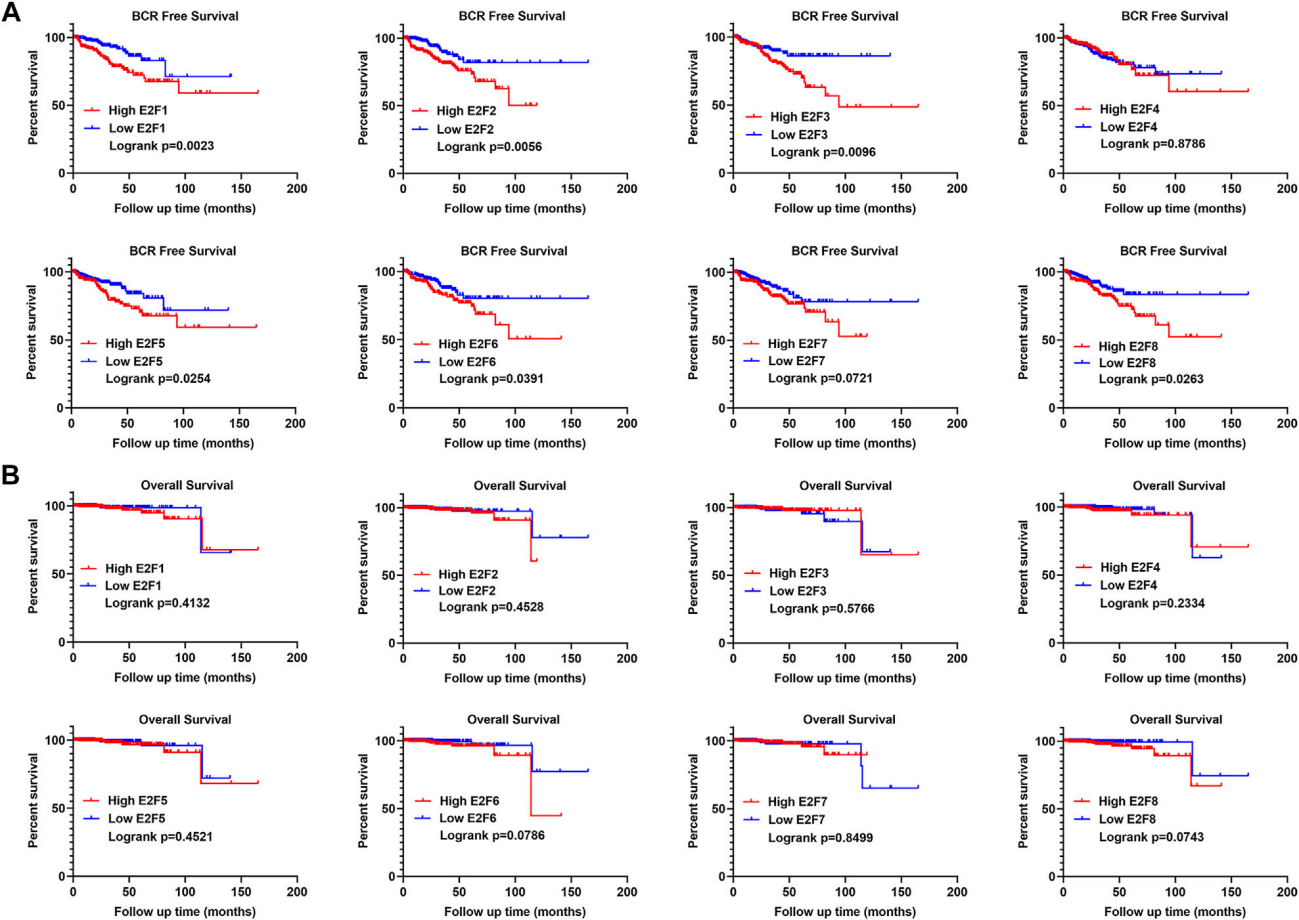
Prognostic value of E2Fs in patients with prostate cancer. **(A)** BCR-free survival curves in prostate cancer patients with high and low expressions of E2Fs. **(B)** Overall survival curves in prostate cancer patients with high and low expressions of E2Fs.

In addition, we performed a survival analysis of the eight E2Fs in patients with PCa. Patients were divided into two groups based on the median expression level of E2Fs in PCa. The results indicated that patients with a higher expression of E2F1, E2F2, E2F3, E2F5, E2F6, and E2F8 had a higher risk of biochemical recurrence (BCR) (Figure 3A, log rank *p* < 0.05). There was no significant difference in the overall survival of patients in groups based on the eight E2Fs (Figure 3B). Overall, E2F1, E2F2, E2F3, and E2F5 were found to be correlated with the malignant progression of prostate cancer.

**FIGURE 4:**
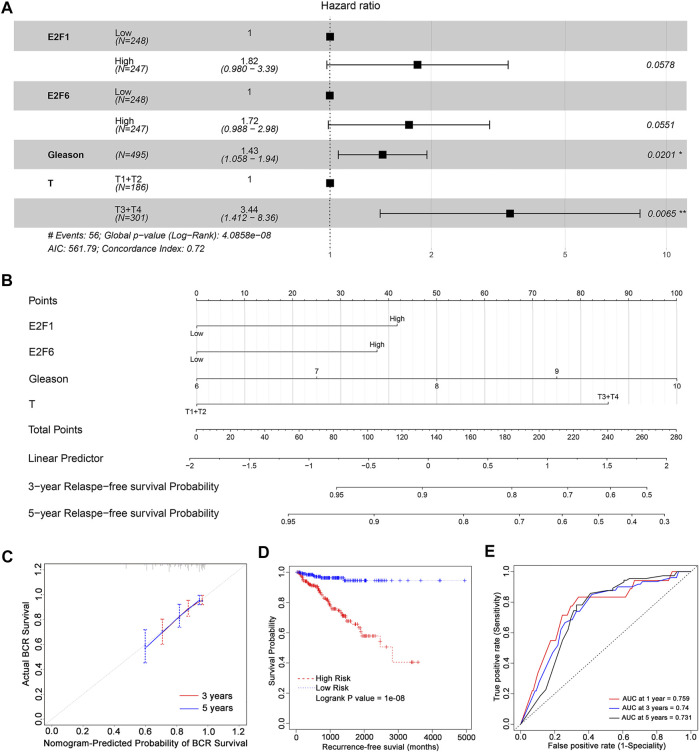
The prognostic value of E2F expression combined with clinical features in prostate cancer. **(A)** Stepwise Cox regression analysis of E2Fs, Gleason score, and pathological tumor stage in the TCGA-PRAD cohort. Nomograms **(B)**, including the calibration plots **(C)** for the prediction of relapse-free survival (RFS) for PCa patients at 3 and 5 years. **(D)** BCR-free survival curves in prostate cancer patients with high and low risk scores. **(E)** AUC curves for the ability of the risk score to predict 1-, 3- and 5-year RFS in prostate cancer patients.

To further examine the prognostic value of the E2F family in prostate cancer, a stepwise regression analysis was performed using the TCGA-PRAD dataset. As shown in Figure 4A, the results revealed that E2F1 (*p* = 0.0578), E2F6 (*p* = 0.0551), Gleason score (*p* = 0.0201), and tumor stage (*p* = 0.0065) were correlated with the disease-free survival of prostate cancer, though the *p* values of E2F1 and E2F6 were slightly below the cut-off for significance.

Based on the significant prognostic value of E2F1 and E2F6 as well as their association with tumor stage and Gleason score in prostate cancer, we generated nomograms for predicting a patient’s 1-year, 3-year and 5-year relapse-free survival (Figure 4B). The calibration curves in Figure 4C show that these nomograms had a similar ability to predict outcomes to an ideal model (diagonal line).

In addition, we split patients into high-risk and low-risk groups using the median risk score. Patients with high risk scores had poorer outcomes than those with low risk scores (Figure 4D, log rank P = 1e-08). ROC analysis indicated that the AUCs of the nomograms were 0.759, 0.74 and 0.731 for 1-year, 3-year and 5-year relapse-free survival, respectively (Figure 4E). These AUC values of the nomograms suggested that our model for evaluating the prognosis of patients with prostate cancer is a novel model that can be used for the early, accurate judgment of prognosis and application of interventions.

### Analysis of Somatic Mutations in Different Subgroups Based on Clinicopathologically Significant E2Fs

**FIGURE 5:**
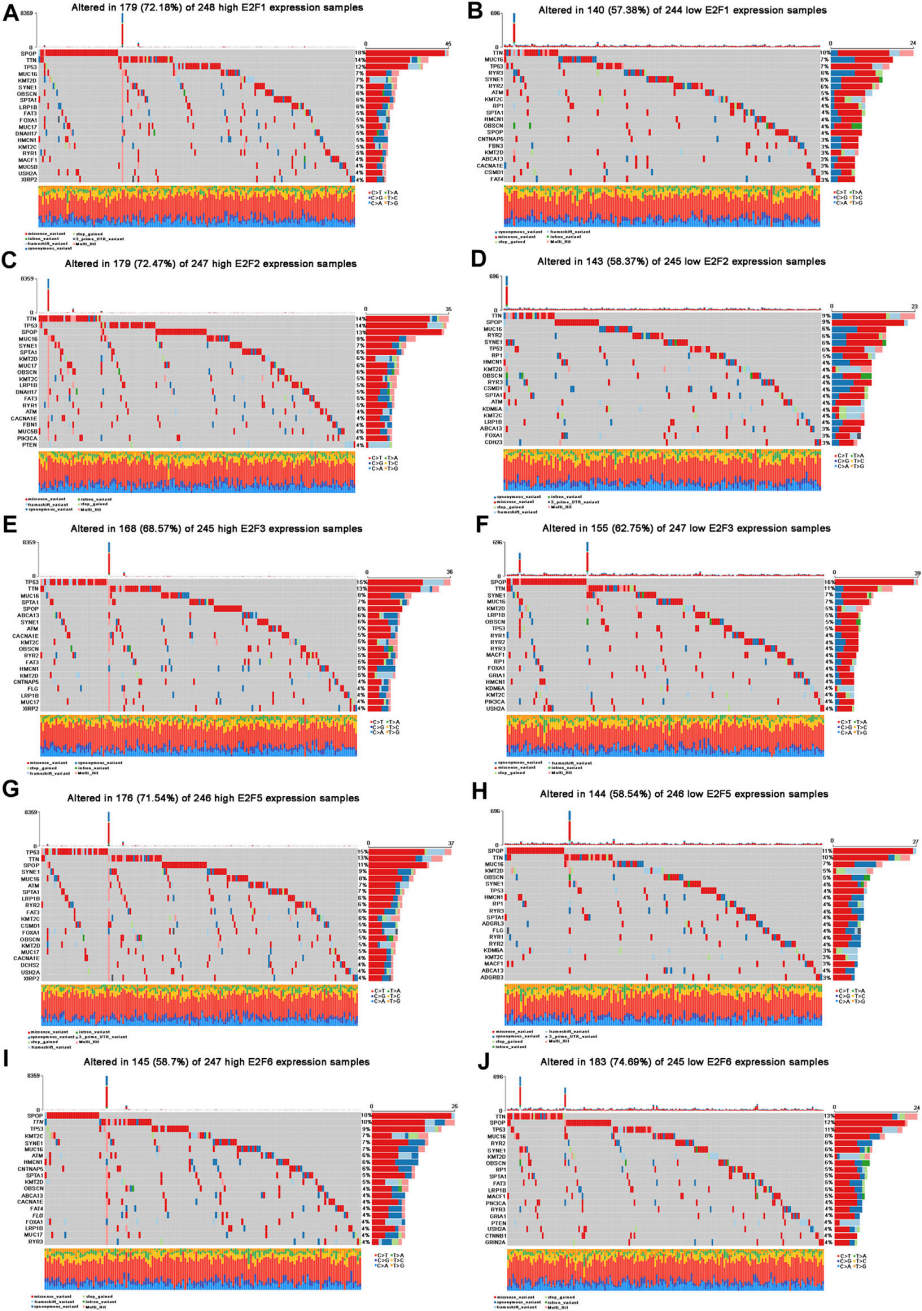
Landscape of mutation profiles in PCa patients with an aberrant expression of E2Fs. **(A–J)** Waterfall plots represent mutation information in each PCa patient sample in the high and low E2F1, E2F2, E2F3, E2F5, and E2F6 expression groups.

Given that prostate cancer is a highly heterogeneous tumor, somatic mutations of clinicopathologically significant E2Fs, including the E2F1, E2F2, E2F3, E2F5, and E2F6, were further investigated in PCa patients. Based on their median gene expression levels, PCa patients were divided into a high expression group and a low expression group. Mutation information for each gene in each sample was visualized with waterfall plots (Figure 5).

The top 10 mutated genes in the high E2F1 expression group were SPOP, TTN, TP53, MUC16, KMT2D, SYNE1, OBSCN, SPTA1, LRP1B, and FAT3 (Figure 5A), while the top 10 mutated genes in the low E2F1 expression group were TTN, MUC16, TP53, RYR3, SYNE1, RYR2, ATM, KMT2C, RP1, and SPTA1 (Figure 5B). In the high E2F2 expression group, the top 10 mutated genes were TTN, TP53, SPOP, MUC16, SYNE1, SPTA1, KMT2D, MUC17, OBSCN, and KMT2C (Figure 5C). In addition, TTN, SPOP, MUC16, RYR2, SYNE1, TP53, RP1, HMCN1, KMT2D, and OBSCN were the top 10 mutated genes in the low E2F2 expression group (Figure 5D). In the E2F3 group, the top 10 mutated genes in the high expression group were TP53, TTN, MUC16, SPTA1, SPOP, ABCA13, SYNE1, ATM, CACNA1E, and KMT2C (Figure 5E), and the top 10 mutated genes in the low expression group were SPOP, TTN, SYNE1, MUC16, KMT2D, LRP1B, OBSCN, TP53, RYR1, and RYR2 (Figure 5F). Furthermore, the top 10 mutated genes in the high E2F5 expression group were TP53, TTN, SPOP, SYNE1, MUC16, ATM, SPTA1, LRP1B, RYR2, and FAT3 (Figure 5G), while the top 10 mutated genes in the low E2F5 expression group were SPOP, TTN, MUC16, KMT2D, OBSCN, SYNE1, TP53, HMCN1, RP1, and RYR3 (Figure 5H). Moreover, SPOP, TTN, TP53, KMT2C, SYNE1, MUC16, ATM, HMCN1, CNTMAP5, and SPTA1 were the top 10 mutated genes in the high E2F6 expression group (Figure 5I). The top 10 mutated genes in the low E2F6 expression group were TTN, SPOP, TP53, MUC16, RYR2, SYNE1, KMT2D, OBSCN, RP1, and SPTA1 (Figure 5J).

### The Associations Between Different E2Fs and Tumor Immune Components

**FIGURE 6:**
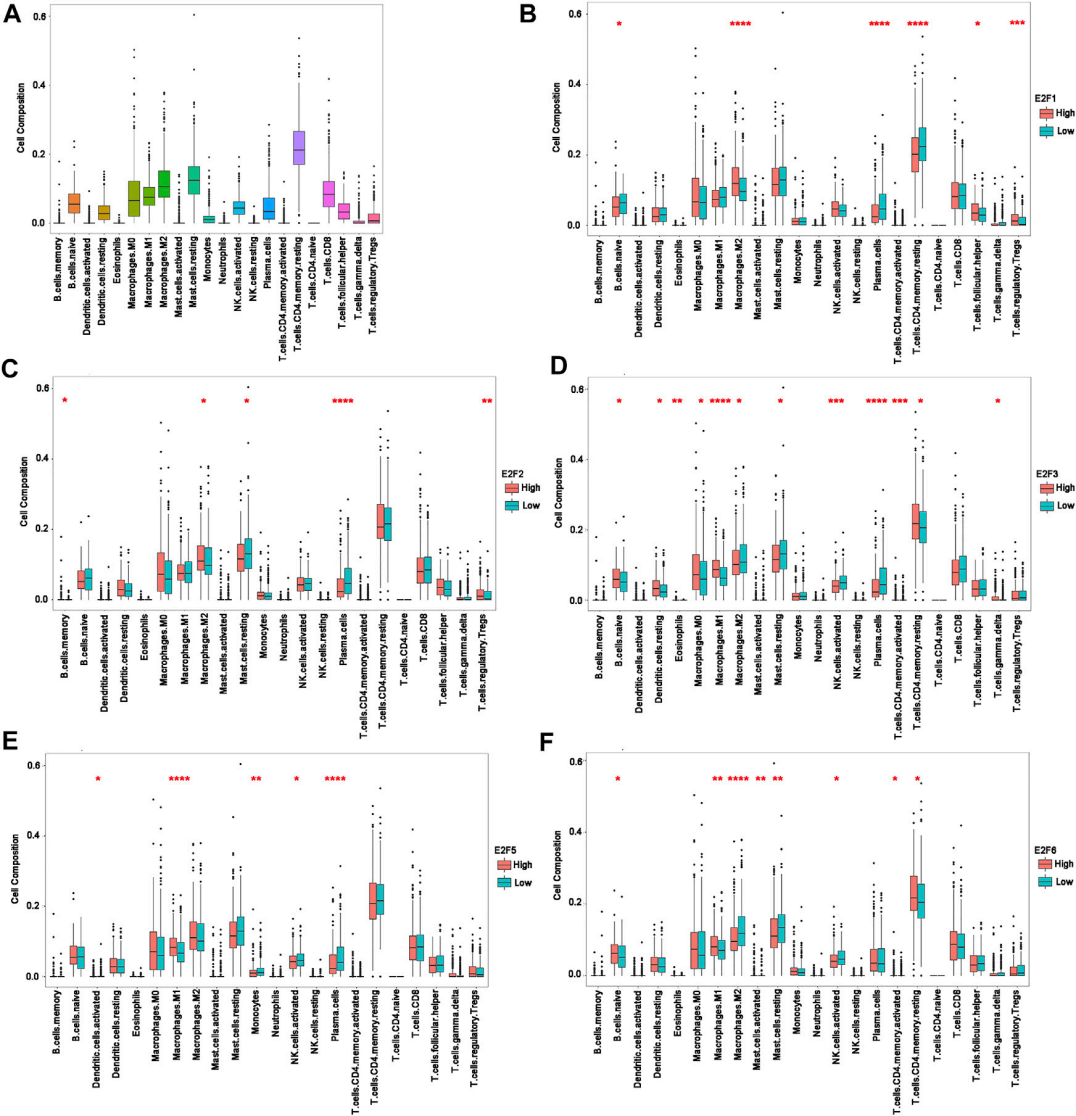
Intratumoral immune cell composition analysis. **(A)** The proportions of 22 immune cell types in prostate cancer from TCGA datasets. **(B–F)** The boxplot shows the different levels of 22 infiltrating immune cell types in groups with high and low E2F1, E2F2, E2F3, E2F5, and E2F6 expressions. *p < 0.05; **p < 0.01; ***p < 0.001; ****p < 0.0001.

Analysis of the TCGA dataset revealed that certain immune cell types, such as M2 macrophages, resting mast cells, and resting CD4^+^ memory T cells, were abundant in PCa. (Figure 6A). Subsequently, we further investigated the alteration of immune cell components in samples with altered expressions of E2F1, E2F2, E2F3, E2F5, and E2F6 in prostate cancer based on TCGA.

The infiltration levels of M2 macrophages were decreased in patients with a low expression of E2F1 and E2F2 and were increased in patients with a low expression of E2F3 and E2F6. In contrast, patients with a higher expression of E2F3, E2F5, and E2F6 had higher M1 macrophage infiltration, while the opposite was observed with activated NK cells. In regard to resting mast cells, higher infiltration levels were observed with a lower expression of E2F2 and E2F3. Increased infiltration of plasma cells was found in the samples with lower E2F1, E2F2, E2F3, and E2F5 gene expressions.

Additionally, the expression of E2F1 was directly correlated with the infiltrating levels of follicular helper T-cells and regulatory T-cells, and negatively correlated with the infiltrating levels of resting CD4^+^ memory T-cells and naive B-cells. More memory B-cells and regulatory T-cell infiltrates were observed in samples with higher E2F2 levels. Furthermore, the expression of E2F3 was significantly related with the infiltrating levels of naive B-cells, resting dendritic cells, eosinophils, M0 macrophages, activated CD4^+^ memory T cells, resting CD4^+^ memory T-cells, and gamma and delta T cells. In patients with E2F5 alterations, the lower the expression levels of E2F5 were, the more abundant the proportions of activated dendritic cells and monocytes. Lastly, the levels of infiltrating naive B cells, activated CD4^+^ memory T cells, resting CD4^+^ memory T cells and activated mast cells were positively related to E2F6, while resting mast cells showed a negative correlation with E2F6 (Figures 6B–F).

### Analysis of Enriched Pathways and Functions Based on E2Fs in Patients With Prostate Cancer

**FIGURE 7:**
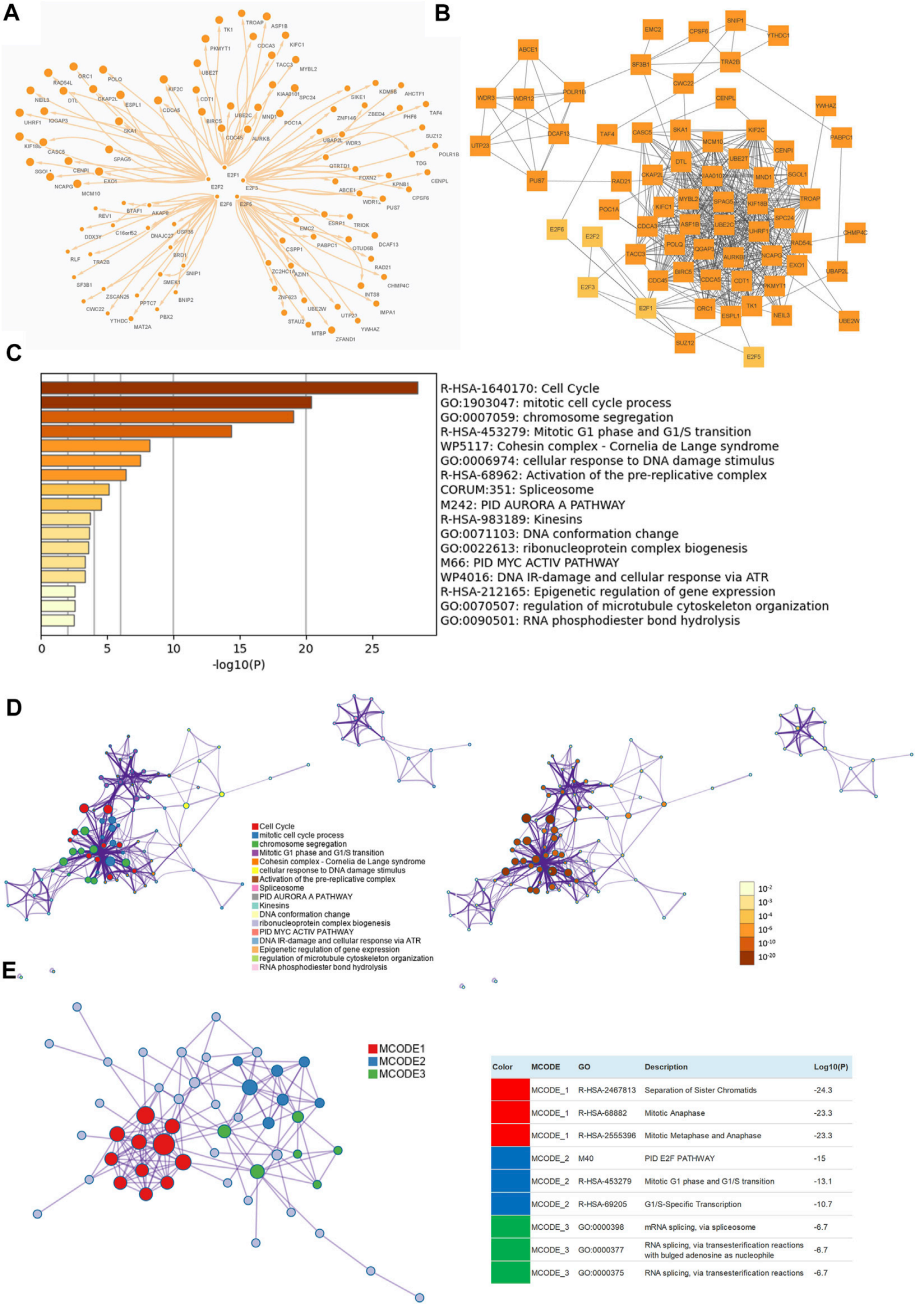
Functional enrichment analysis of E2Fs and neighboring genes in prostate cancer. **(A)** The top 20 related genes of E2F1, E2F2, E2F3, E2F5, and E2F6 in TCGA-PRAD. **(B)** Protein–protein interaction (PPI) network among top E2F-related genes made by STRING. **(C)** Bar graph of the enriched terms across E2F genes colored by *p* values. **(D)** Network of enriched GO terms colored by cluster ID (left) and by *p* value (right). **(E)** The three most significant MCODE components from the PPI network (Metascape).

To further understand the potential mechanism of E2Fs in the progression of prostate cancer, E2F1-, E2F2-, E2F3-, E2F5-, and E2F6-related genes in samples from TCGA-PRAD were assessed by functional enrichment analysis. The top 20 related genes of E2F1, E2F2, E2F3, E2F5, and E2F6 were selected (Figure 7A). Using the STRING protein–protein interaction analysis tool, 58 co-expressed genes were found to interact with each other (Figure 7B).

In Metascape, the functions of these interacting genes were examined. The top 17 clusters with their representative enriched terms were visualized (Figure 7C). E2F1, E2F2, E2F3, E2F5, and E2F6 and their neighboring genes were mainly enriched in terms such as cell cycle, mitotic cell cycle process, chromosome segregation, mitotic G1 phase and G1/S transition, cohesin complex, activation of the pre-replicative complex, spliceosome, PID, AURORA A pathway, and kinesins.

**FIGURE 8:**
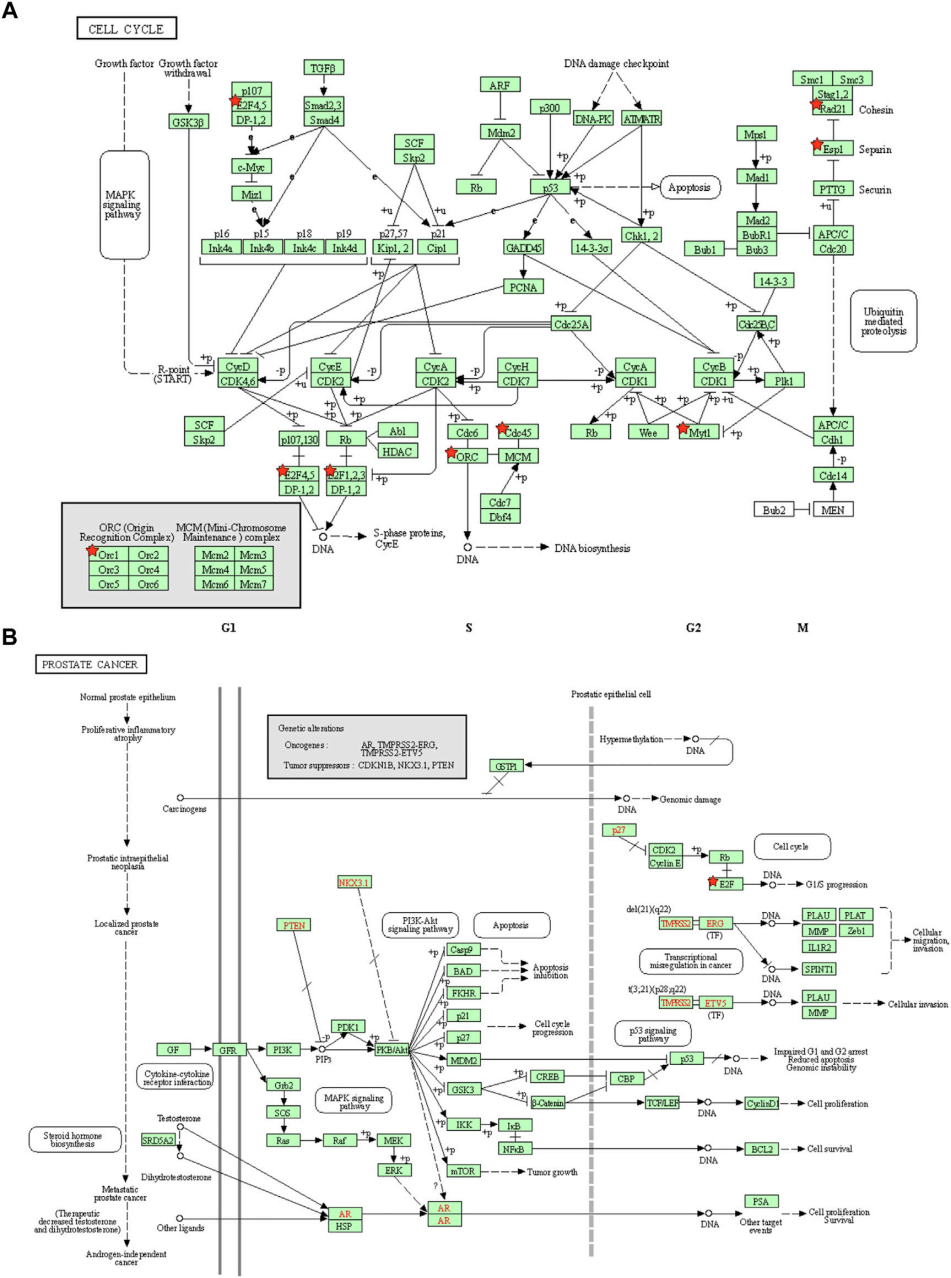
Cell cycle and prostate cancer pathways regulated by the altered E2Fs in prostate cancer. **(A)** Cell cycle pathways regulated by the altered E2Fs in prostate cancer. **(B)** Prostate cancer progression pathways regulated by the altered E2Fs in prostate cancer.

Furthermore, the network was visualized using Metascape, and each enriched term was presented as a node and was colored first by its ID (Figure 7D left) and subsequently by its *p* value (Figure 7D right). A protein–protein interaction enrichment analysis was also carried out to better understand how E2F family members work. The protein–protein interaction network and MCODE components identified in the gene lists are shown in Figure 5E. Finally, a KEGG analysis was used to elucidate the molecular mechanisms of the E2F family and its surrounding genes, and the results indicated that these genes mainly participated in cell cycle processes in PCa (Figures 8A,B).

## Discussion

Prostate cancer (PCa) has long been known to be a heterogeneous disease. Lethal metastatic prostate cancer seems to arise from a single clone in the primary tumor but can exhibit subclonal heterogeneity at the genomic, epigenetic, and phenotypic levels (Haffner et al., 2021). This complex tumor heterogeneity contributes to the limited utility of diagnostic and prognostic indicators for prostate cancer patients (Silberstein et al., 2013; Shoag and Barbieri, 2016; Whitaker et al., 2020).

Numerous studies have revealed that the aberrant expression of E2Fs is linked to a variety of malignancies, including pancreatic cancer, breast cancer, and glioblastoma (Peng et al., 2017; Iino et al., 2020; Yang et al., 2020). Comprehensive analyses of the E2F family in breast cancer, lung cancer, and ovarian cancer have also been carried out (Sun et al., 2018; Sun et al., 2019; Zhou et al., 2019). Compared to a study that also explored the functional and prognostic roles of E2Fs in human prostate cancer (Wang et al., 2021), we further conducted a Kaplan-Meier analysis using biochemical recurrence (BCR)-free survival, which is a critical index to judge the prognosis of patients with PCa, and established a novel predictive prognostic model that combines E2F1, E2F6, Gleason score, and clinical stage and may be useful for future clinical practices. Of importance, our study also predicted the function of the associations between somatic mutations and infiltrating immune cells in different E2F subgroups, suggesting that specific E2Fs may be targetable in combination with immunotherapy in prostate cancer.

Based on public transcriptome sequencing data, we systematically explored the expression patterns, prognostic value, genetic alterations, correlations, and potential functions of different E2Fs in PCa. Additionally, we analyzed the relationships between E2F subtypes and clinical characteristics of patients with PCa. In this study, E2F1, E2F2, E2F3, and E2F5 were shown to be up-regulated in the malignant prostate tissue compared to the benign tissue, and their up-regulation was correlated with advanced stage and poor biochemical recurrence survival in PCa patients. Although there was no significant difference in E2F6 expression between patients with different Gleason scores and clinical stages, a high E2F6 expression was related to metastasis and biochemical recurrence. In summary, E2F1, E2F2, E2F3, E2F5, and E2F6 were found to be correlated with the malignant progression of prostate cancer.

Although E2F4, E2F7, and E2F8 did not show a significant effect on either diagnosis or prognosis, some studies have reported that activated E2F4 can minimize the proliferation of PCa cells in response to radiation (Crosby et al., 2007) and is involved in the progression of PCa (Yang et al., 2008). E2F7 is believed to promote tumor progression in various cancers as the target of miRNA-302a/d (Ma et al., 2018). In regard to E2F8, it has been reported that geraniol can suppress prostate cancer growth by down-regulating E2F8 (Lee et al., 2016). In short, few studies have focused on the function of these three E2Fs in PCa, and the roles of E2F4, E2F7, and E2F8 in prostate cancer remain to be explored.

The underlying mechanism of the cell cycle pathway in the progression of cancer has been widely studied. The significant E2Fs and their correlated genes were also mainly enriched in pathways related to the cell cycle. In previous studies, E2F1 has been validated to play an important role in the cell cycle and in proliferation, apoptosis, and differentiation (Chen et al., 2009; Lee et al., 2010; Liang et al., 2016). In PCa, E2F1 is reported to be driven by POM121 (Rodriguez-Bravo et al., 2018) and cooperates with miR-20b-5p and TGFBR2 to form a regulatory loop to participate in epithelial to mesenchymal transitions in PCa (Qi et al., 2019).

To our knowledge, few studies about the relationship between E2F2 and the cell cycle pathway in PCa have been reported to date. However, an altered expression of E2F2 was reported to be associated with PTEN-mediated G1 cell cycle arrest in LNCaP cells (van Duijn et al., 2010), and this altered expression may be regulated by miR-31 and involved in the disruption of androgen receptor homeostasis in PCa (Lin et al., 2013).

Consistent with our study, E2F3 was found to be over-expressed in PCa and to stimulate the proliferation of prostate cancer cells. A high expression level of E2F3 was also found to be an independent factor for poor patient survival (Foster et al., 2004; Olsson et al., 2007). An increased E2F3 protein level in PCa can contribute to a decrease in miR-34c expression, as reported by Hagman et al. (2010). Additionally, E2F3 was found to directly target the interleukin six receptor in Pca-derived cells (Libertini et al., 2012). E2F3-dependent transcription and cellular transformation are mediated by the SNF2-like helicase HELLS in Pca (von Eyss et al., 2012). However, the specific regulatory relationship between E2F3 and cell cycle pathways has yet to be studied.

E2F5 is considered to be an oncogene in PCa. E2F5 targets TFPI2, MMP-2, and MMP-9 and promotes the migration and invasion of PCa cells (Karmakar et al., 2020). Down-regulation of E2F5 by miR-1-3p can lead to the inhibition of prostate cancer cell aggressiveness *in vitro* (Li et al., 2018). Conversely, up-regulation of E2F5 enhances CDK13 transcription and promotes circCDK13 biogenesis, which in turn relieves the repression of E2F5 expression, subsequently promoting the expression of E2F5 and PCa cell proliferation (Qi et al., 2021).

Similar to E2F2 and E2F4 mentioned previously, E2F6 has been reported in few prostate cancer studies. Zhang et al. (2014) demonstrated that an increased expression of miR-31 could decrease E2F6, resulting in the sensitization of prostate cancer cells to docetaxel-induced apoptosis. Similarly, miR-205 and miR-31 promote chemotherapeutic agent-induced apoptosis of prostate cancer cells by down-regulating Bcl-w and E2F6 (Bhatnagar et al., 2010). Overall, little is known about the role of E2F6 in PCa.

To further investigate the clinical prognostic value of E2Fs in PCa patients, a stepwise regression analysis was performed, and E2F1 and E2F6 were identified to predict the disease-free survival of prostate cancer together with Gleason score and tumor stage. Using the prognostic model established by these screening factors, we could accurately identify the population at a high-risk of biochemical recurrence in patients with PCa. In addition, Liu et al. (2019) reported that PARP inhibition could suppress GR-MYCN-CDK5-RB1-E2F1 signaling and neuroendocrine differentiation in CRPC, which means that the combination of E2F1 and E2F6 could be a new biomarker for early and accurate treatment intervention. This result suggests the clinical applicability of our prognostic model.

Mutations in gene sets often cause tumorigenesis and induce tumor heterogeneity. After deeply analyzing the somatic mutations in different subgroups of E2Fs, we found that FAT atypical cadherin 3 (FAT3) was mutated more frequently in samples with a higher expression of E2F genes (E2F1: 5%, E2F2: 5%, E2F3: 5%, E2F5: 6%). FAT3 is known to be related to calcium binding and the regulation of cytoskeletal organization (Marcinkevicius and Zallen, 2013). Shared processes that are critical for tumor growth and Ca^2+^ signaling within cancer cells have been well investigated (Monteith et al., 2017). Our results reveal for the first time that E2Fs may regulate the cell cycle through the calcium signaling pathway.

Prostate cancer is a heterogeneous disease, and tumor immune components are altered accordingly. Thus, we estimated the levels of infiltrating immune components by using CIBERSORT. The immune microenvironment and immunotherapy in cancer have been investigated in multiple studies and clinical trials (von Rundstedt and Necchi, 2017). Our analysis of immune components revealed that plasma cells and NK cells were more abundant in samples with a lower expression of E2F1, E2F2, E2F3, and E2F5. Plasma cells have been reported to activate NK cells in the tumor microenvironment by producing large amounts of antibodies that can promote antitumor immunity by driving antibody-dependent cellular cytotoxicity (ADCC) (Sharonov et al., 2020). Given the results of our immune infiltration analysis, we propose that E2F1, E2F2, E2F3, and E2F5 may potentially affect the progression of cancer through anticancer immunity.

In conclusion, we identified E2F1 and E2F6 as new important indicators that can be used in combination with commonly used clinical indicators to assess the risk of BCR in patients with PCa and explored potential mechanisms regulated by the E2F family. We believe that our findings will enrich existing knowledge and therapeutic approaches and, importantly, enhance the prognostication accuracy for PCa patients.

## Data Availability

The datasets presented in this study can be found in online repositories. The names of the repository/repositories and accession number(s) can be found in the article/Supplementary Material.
